# The Importance of Parents’ Behavior in their Children’s Enjoyment and Amotivation in Sports

**DOI:** 10.2478/hukin-2013-0017

**Published:** 2013-03-28

**Authors:** Pedro A. Sánchez-Miguel, Francisco M. Leo, David Sánchez-Oliva, Diana Amado, Tomás García-Calvo

**Affiliations:** 1 Faculty of Teaching Education, University of Extremadura.; 2 Faculty of Sports Science, University of Extremadura.

**Keywords:** team sports, amotivation, athletes, family

## Abstract

The main aim of the research was to examine the relationship between motivational orientations and parents’ behavior with regard to the players’ motivational orientation, motivational climate, enjoyment and amotivation. The sample comprised 723 athletes (M = 12.37, SD = 1.48) and 723 parents (M = 46.46, SD = 2.56). Players were male and female who belonged to federative basketball, handball, football and volleyball teams. Parents and athletes completed questionnaires that assessed motivational orientations, parents’ involvement in the practice as well as enjoyment and motivation in the sport. Results showed a positive relationship between parents’ support of the sport and players’ enjoyment and a negative relationship with players’ amotivation. Moreover, in players who perceived more pressure from their parents, there was a positive association with amotivation and a negative one with enjoyment. Lastly, it was emphasized that appropriate parental participation can promote an increase of players’ enjoyment of and motivation for sport.

## Introduction

Socialization into sport and physical activity can be considered a modeling process in which family members are powerful role models. Most of the studies revealed that both parents’ exercise patterns and encouragement have an effect on children’s exercise behavior, and that physically active parents tend to have physically active children (Sallis et al., 1999). Thus, youth sport experiences can provide opportunities for personal growth and development that extend beyond the physical domain to athletes’ psychological processes (Boixadós et al., 2004; Müller and Sternad, 2004).

In accordance with this, Horn and Horn (2007) explain that parents’ belief and value systems (e.g., their beliefs, attitudes, values…) determine their behaviors toward their child. The behaviors (e.g., modeling, providing opportunities, emotional support…) then influence the child’s belief and value systems, which determine the child’s behavior. In other words, most young athletes believe that their parents provide them with a supportive, stable, secure and encouraging environment (Helldsted, 1995). Knight et al. (2010) conducted a research with 42 Canadian tennis players aged from 12 to 15. This study was meant to identify the way athletes would like their parents to behave during competition. Data analysis uncovered one dominant theme: the players wanted their parents to be involved in and to support their sporting experience.

Therefore, instead of behavior that pressures players to perform and succeed, they would prefer supportive comments about the positive aspects of their attitude, sportsmanship and effort. Furthermore, unless their parent had legitimate experience with their sport, the athletes did not want technical or tactical coaching from them. However, they were happy to receive practical advice. The athletes were also aware of the occasions when their parent’s verbal comments were not consistent with their nonverbal signals, and of what their body language communicated (Horn and Horn, 2007).

Furthermore, Holt et al. (2008) found that parents’ verbal reactions to their children’s performance ranged on a continuum from supportive to more controlling comments, including remarks such as praise/encouragement, instruction, and derogatory remarks. Regarding parental pressure, Anderson et al. (2003) pointed out that as parental pressure increased, children’s reported enjoyment and satisfaction decreased. Parents may believe that expressing disappointment regarding a child’s poor performance will promote the motivation for improvement, but from the child’s perspective, even well meant parental pressure can backfire and may contribute to a child’s lowered enjoyment and motivation. Additionally, excessive parental pressure has been linked to athlete’s perceived negative affect. To avoid placing excessive pressure on a child, it is crucial to be realistic about what they can and want to accomplish in the sport (Hellstedt, 1995). According to this, Lee and McLean (1997) studied the perceptions of parental pressure in adolescent swimmers and reported that pressure was associated with perceived directive or controlling behaviors by parents. Youths’ perceptions of their parents’ attitudes and behaviors concerning the sport were associated with self-perceptions of ability, motivational orientation and attitudes and behaviors in sport and physical education (Brustad et al., 2001; Fredricks and Eccles, 2004). For instance, family participation and an active involvement in the children’s physical activity promote players’ greater satisfaction and positive participation in their sport career (Torregrosa et al., 2007; Wuerth et al., 2004). Consequently, children’s motivation to participate in sport is a key consideration for researchers, coaches and parents (Keegan et al., 2009). Basically, if we want to engage children in sport practice from an early age and progress to reach their full potential, then it is definitely essential to have a good environment during these formative years, and parents play a very important role in this process.

Therefore, motivation has emerged as a potential factor that contributes to promote an appropriate physical activity (Weiss and Ferrer-Caja, 2002). Thus, it would be interesting to determine how family behavior influences motivational aspects and other crucial psychological variables for appropriate sport practice (Gould et al., 2006; 2008). In this regard, most of the studies have not directly measured significant others (e.g., family, peers, coaches…), but they have assessed participants’ perception of their coaches (Olympiou et al., 2008), peers (Smith et al., 2006) or parents (Papaioannou et al., 2008). At the same time, this research reveals new information about the relationships between parents and athletes in sport practice, and aims to examine the influence of parents’ behaviors on their children’s adaptive or maladaptive behaviors. This assessment was carried out using parents matched with their own children. This study is therefore unique because only a few works in the sport domain have analyzed the psychological aspects of both groups. Another important aspect of this work is that only a few works in the scientific literature have directly examined parents’ perceptions of their children’s motivation.

Given the state of the literature and the concerns about parents in youth sports, the main aim of this study was to examine the relationship between parents’ motivational orientation and behaviors in their influence on youth players’ orientation and motivational climate, enjoyment, and motivation. As a first hypothesis, it was proposed that parents’ task orientation and motivational climate would be positively related to their children’s task orientation and motivational climate. The second hypothesis stated that parents’ pressure would be negatively associated with their children’s enjoyment and positively related to their amotivation.

## Material and Methods

### Participants

The sample comprised 723 athletes and 723 parents. Of the parents, 351 were mothers and 372 fathers, ranging in age from 36 to 49 years old (*M* = 46.46, *SD* = 2.56). The players were male (*n* = 561) and female (*n* = 162), ranging in age between 11 and 16 years old (*M* = 12.37, *SD* = 1.48). All players belonged to federative basketball, handball, football and volleyball teams and held a federative card with their personal and sports data. The methodology used in this study was described elsewhere (Nuviala et al., 2011). Briefly, the populations were selected by multi-step, simple random sampling—first taking into account the population from Extremadura (Spain) and then by random assignment of the club within each city. The cities and clubs were chosen according to their involvement and commitment to participate in the study, their geographical location in the region (north – south gradient in order to be representative), and taking into account the main researcher’s possibility to travel to that city.

### Measures

#### Motivational orientations

The Spanish version (Cervelló et al., 1999) of the Perception of Success Questionnaire (POSQ: Roberts et al., 1998) was used to measure the young participants’ goal orientations in the sport. The questionnaire consists of 12 items. Six of them correspond to the athletes’ “task orientation” factor (e.g., “I feel I am successful in sport if I work hard”), and the remaining six correspond to their ‘ “ego orientation” factor (e.g., “I feel I am successful in sport if I beat the others”). To assess parents’ motivational orientation, we used an adapted version of this instrument, modifying the original introductory sentence as follows: “As a parent, I have a sense of success when my son/daughter…” Reliability (internal consistency) of the motivational orientation measures was assessed, and each measure attained a Cronbach alpha between .77 and .85.

#### Parents’ involvement in sport practice

To measure parents’ involvement, we employed a Spanish version of the Parents’ Involvement Sport Questionnaire (PISQ: Wurth et al., 2004; Lee and Mclean, 1997) previously applied by Torregrosa et al. (2007). This questionnaire has 20 items divided into four main factors: directive behavior (5 items, e.g., “Before the match, your parents tell you how to play”), support and comprehension (6 items, e.g., “Although the game was lost, your parents encourage you because you performed well”), active implication (5 items, e.g., “Your parents speak with your coach about your improvement in the sport”) and pressure (4 items, e.g., “Your parents press you to train better”). To assess parents’ behaviors in the sporting practice, an adapted version was applied, modifying the original introductory stem to: “As a parent, you adopt a behavior towards your child in which…” (e.g., “Before the matches, you tell him/her how to play”, “You press him/her to train better”). Internal consistency revealed values between .77 and .85.

#### Enjoyment of practice

This is one of the five factors forming the Sport Commitment Questionnaire (SCQ) by Scanlan et al. (1993), validated in the Spanish context by Sousa et al. (2007). It has 4 items (e.g., “I enjoy myself practicing sport in this season). Reliability (internal consistency) attained a Cronbach alpha value of .81.

#### Amotivation in sport practice

This is one of the five factors of the Sport Motivation Scale (SMS) by Pelletier et al. (1995), validated in the Spanish context by Balaguer et al. (2007). It has 4 items (e.g., “It is not clear for me anymore; I don’t really think my place is in sport”). The closed response obtained an internal consistency of .89.

Responses to these questionnaires were closed and they were rated on a 5-point Likert scale ranging from 0 (*strongly disagree*) to 5 (*strongly agree*).

### Procedure

The study was carried out using a correlational methodology, that is, we measured parents and athletes at the beginning of the season (from September to November). We developed a protocol to collect similar data from all the participants involved in the research. Prior to participation in the study, the general purpose of the study was explained to coaches and parents, and permission to participate in the study was requested from them.

The athletes completed the questionnaires in the changing room, and this took approximately 15–20 minutes. The main researcher was always present and encouraged the athletes to ask questions as needed. They also were asked to answer the questions as honestly as possible and were reassured that their responses would remain strictly confidential.

Regarding parents, it is important to note that questionnaires were only answered by the person (father or mother) who was more involved in the children’s’ sporting practice. To determine who was the most involved, we asked the participant athletes who brings them to training sessions, who watches the games and shows more interest in the practice and who was a model to follow. After determining who the most involved person was, we gave a questionnaire to each athlete to take home and be completed by this person, asking them to bring it back the next training session. To complete data collection, we matched parents and children before including them in the database. That is, the father/mother of each athlete was in the database.

### Analysis

Data was analyzed using the SPSS 19.0 statistical package. Firstly, various tests were conducted to determine the nature of the data. We used the K-S test for independent samples to verify the normality of the groups, the runs test for randomness, and Levene’s test for the homoscedasticity. As the data were parametric, we applied the following parametric tests to analyze the data: reliability analysis, descriptive analysis, bivariate correlations, and linear regression analysis.

With regard to the validity of the instruments, we obtained an adequate structure of each scale through factorial analysis. Moreover, as mentioned, in the reliability analysis, all the factors attained a Cronbach alpha level over .70, which is considered acceptable.

## Results

### Descriptive statistics

Table 1 shows the descriptive statistics of the variables of the study. The scores obtained in the athletes’ and parents’ motivational orientations were similar, with lower values in ego orientation than in task orientation.

The results in the variables athletes’ enjoyment and perception of their parents’ support obtained higher means than their amotivation and perception of parents’ pressure. Likewise, the variables of parents’ support and involvement obtained higher mean values than pressure and directive behavior. Thus, athletes and parents both have lower levels in the variables with negative connotations than in the variables that imply positive behaviors. Kurtosis and skewness scores were acceptable.

### Correlational Analysis

Table 2 shows the correlations between the diverse variables of the study. There was a positive relationship between parents’ motivational orientation and their children’s orientation, and this association was stronger when their motivational orientations were the same. It is noteworthy that directive behavior and pressure were positively related to ego orientation, and parents’ support was related to task orientation.

Regarding the relationships between parents’ behaviors and children’s perception of those behaviors, stronger correlations were found between the same categories as perceived by players and their parents, with values over .30.

Lastly, athletes’ enjoyment and amotivation had an important relationship with parents’ behavior in the sporting practice. Thus, when parents admitted pressuring their children in the sport, the children’s amotivation was greater and their enjoyment in the practice was lower. On the other hand, parents’ support was positively correlated with athletes’ enjoyment and negatively associated with their amotivation.

### Regression Analysis

To determine the set of variables that help to predict enjoyment and amotivation in the sport, a stepwise regression analysis was conducted, using enjoyment and amotivation as dependent variables, and parents’ orientations and behaviors as independent variables.

Table 3 shows that parents’ support was the strongest predictor of athletes’ enjoyment in the first step, accounting for 16 % of the variance. In this case, this variable positively predicted enjoyment, that is, the greater the parents’ support, the higher will be the young athletes’ enjoyment of the sport. In the second step, parents’ pressure was entered, explaining an additional 9% of the variance. However, in this case, the prediction was negative: the greater the parents’ pressure, the less their children will enjoy the sport.

When we use amotivation as the dependent variable, only parents’ pressure was a predictor, explaining 12 % of the variance of amotivation. The relationship was negative: the greater the parent’s pressure, the higher their children’s amotivation in the sporting practice.

## Discussion

The main goal of the study was to examine the relationship between athletes’ motivational orientations and parents’ behaviors, as well as parents’ influence on their children’s motivational orientations, enjoyment and amotivation. Although parents’ influence is not questioned, this domain of research remains limited, and some of the processes by which this influence may occur still need to be clarified. More particularly, recent reviews of the literature pointed out the lack of information about parents, the scarcity of works carried out directly with parents of young athletes, and the fact that parents’ behaviors were not clearly related to specific children’s outcomes, although the focus is on relatively young samples, leaving a gap in our knowledge about this influence (Horn and Horn, 2007).

The first hypothesis proposed that parents’ task orientation would be positively related to children’s task orientation, as well as to their motivational climates. In this sense, the global scores revealed a significant and positive relationship between children’s orientations and motivational climates and their parents’ orientations and climates. That is, the greater the parents’ dispositional task orientation, the greater was this motivation in their children. Previously, some authors had already highlighted the parents’ role as the greatest and most important influence in the creation of their children’s motivational orientation (Carr and Weigand, 2002). In accordance with this, Harter (1981) showed that parents play an important role in their children’s development, both in the academic domain (Bois et al., 2005) and in the sport domain (Horn and Horn, 2007).

For instance, the outcomes revealed similar relationships between parents’ behaviors and players’ perception of these behaviors, indicating that the strongest correlation occurred among the same behaviors perceived by players and parents. Furthermore, it is relevant that parents’ behaviors are associated with their children’s motivational orientations (Brustad et al., 2001; Fredricks and Eccles, 2004). However, when parents’ employed greater directive behavior and pressure, players showed a dispositional ego orientation, whereas when parents used supportive behavior, the players displayed a dispositional task orientation. Hence, parents’ influence is assumed to be a significant element in the development of young participants’ goal achievement motivation (Roberts, 2001). Taking the above comments into account, the first hypothesis is confirmed, verifying that parents’ motivational issues are related to their children’s motivation through the sport.

The influence of parents’ behaviors did not only appear in motivational aspects (Brustad et al., 2001; Fredricks and Eccles, 2004), but they often affect the youths’ enjoyment of the sporting practice (Brustad, 1996; Scanlan and Lewthwaite, 1986). As can be seen in the results, when the parents considered that they pressed their children in the sport, the children’s amotivation was greater and their enjoyment was lower. These outcomes were corroborated by those found in the regression analysis, where parents’ pressure behaviors negatively predicted the children’s enjoyment and positively predicted their amotivation. Several works have found similar results, where they showed that young people’s opinion of their parents’ involvement was associated with anxiety, amotivation and dropout behavior (Gould et al., 1996; Leff and Hoyle, 1995).

In contrast, parents’ support in training and games was negatively correlated with amotivation and positively associated with players’ enjoyment. Similar outcomes were found by Brustad (1996) and Scanlan and Lewthwaite (1986), who reported positive relationships between parents’ involvement and children’s enjoyment of the sport. Thus, we note that parents’ active involvement (support and comprehension) in the children’s physical activity achieves players’ higher satisfaction and positive participation in the sport (Torregrosa et al., 2007; Hellsted, 1995; Wuerth et al., 2004). In accordance with this, we confirmed the second hypothesis which stated that parents’ pressure would be negatively associated with players’ enjoyment and positively related to amotivation.

Therefore, the main conclusion that we can reach from the study is that in order to motivate and increase children’s enjoyment of sporting practice, it is essential to promote parents’ supportive behaviors and involvement in their children’s school sports, as well as to lower parent’s pressure, thereby decreasing the children’s competitiveness and the emphasis on victory. Diverse strategies can be employed, like the ones used by García-Calvo et al. (2009) or Cruz et al. (2003), who promoted teaching parents so their children could achieve adequate sporting practice, based on positive values, creating prosocial habits and an integral education in the school sport, which allowed the children to develop the sport with pleasure and to keep up their practice for a long time.

In order to reaffirm these conclusions, some limitations of the study should be addressed. In this sense, we highlight that the measurement of orientations and parents’ behaviors was only assessed by the father’s or the mother’s involvement in the sporting practice of their child, and it would be more accurate to measure the behaviors of each parent. Moreover, what was actually measured was the parents’ perception of their behaviors, so it would be also interesting to directly analyze the behavior of parents (e.g., verbal communication, record parents’ performance during training and games…). Finally, this study provides guidelines for parents to optimize their children’s socialization. It is hoped that researchers will continue to examine parental behaviors in sport, and that coaches, athletes will heed the advice of parents and implement their recommendations.

## Figures and Tables

**Table 1:** Descriptive Statistics and Reliability Analysis

*Variables*	*M*	*SD*	*α*	*Kurtosis*	*Skewness*
Athletes					
Ego Orientation	2.95	1.05	.77	−.76	−.07
Task Orientation	4.22	.69	.85	.69	−1.04
Perception of Directive Behavior	2.65	1.05	.78	−.91	−.15
Perception of Parents’ Pressure	1.82	.98	.79	−.96	−.02
Perception of Parents’ Support	4.25	.68	.77	1.36	−1.15
Perception of Parents’ Active Involvement	2.42	1.18	.85	−16	.81
Enjoyment	4.67	.62	.81	1.63	−1.45
Amotivation	1.55	.77	.89	1.46	1.21
Parents					
Ego Orientation	3.04	1.05	.78	1.83	.29
Task Orientation	4.40	.58	.82	1.67	−1.06
Directive Behavior	3.15	1.16	.78	−.49	.40
	2.21	1.11	.79	.73	1.19
Parents’ Pressure					
Parents’ Support	4.12	.81	81	1.73	−1.23
Parents’ Active Involvement	3.05	1.16	.82	−.61	.55

**Table 2:** Correlations among the studied variables

	Athletes’ Ego Orientation	Athletes’ Task Orientation	Athletes’ Perception of Directive Behavior	Athletes’ Perception of Pressure	Athletes’ Perception of Support	Athletes’ Perception of Involvement	Athletes’ Enjoyment	Athletes’ Amotivation
Parents’ Orientation Ego	.24^**^	.12^**^	.01	.06	.04	.04	.04	.05
Parents’ Orientation Task	.06	.25^**^	.05	−.03	.13^**^	.12^**^	.19^**^	−.08
Parents’Directive Behavior	.13^**^	.01	.34^**^	.22^**^	.18^**^	.20^**^	−.00	.07
Parents’ Pressure	.29^**^	−.06	.21^**^	.40^**^	−.04	.11^**^	−.19^**^	.37^**^
Parents’Support	.03	.25^**^	.17^**^	−.04	.39^**^	.25^**^	.31^**^	−.12^*^
Parents’ Involvement	.04	.01	.21^**^	.16^**^	.14^**^	.35^**^	.02	.02

**Table 3:** Regression analysis taking enjoyment and amotivation as dependent variables in the sport

Dependent variable: Enjoyment	*β*	*R^2^*	*t*	*p*
				
Step 1		.16		
Supportive Behavior	.40		12.09	.00
Step 2		.21		
Supportive Behavior	.35		9.46	.00
Pressure	−.19		−5.25	.00

Dependent variable: Amotivation				

Step 1		.12		
Pressure	.34		9.03	.00
